# Engaging Undergraduate Students in Online Data Science Research: Implementation and Impact of a Summer Research Program During COVID-19

**DOI:** 10.3390/educsci16030357

**Published:** 2026-02-25

**Authors:** Laura M. Lessard, Hacene Boukari, Malcolm D’Souza, Heidi H. Kecskemethy, Linda Polasko, Scott Siegel, Erica Singleton, Melinda K. Duncan

**Affiliations:** 1 Delaware IDeA Network of Biomedical Research Excellence (INBRE), University of Delaware, Newark, DE 19716, USA; 2 Delaware State University, Dover, DE 19901, USA; 3 Wesley College, Dover, DE 19901, USA; 4 Nemours Children’s Health, Wilmington, DE 19803, USA; 5 ChristianaCare, Newark, DE 19713, USA

**Keywords:** data science education, online research programs, undergraduate research, remote learning, emergency remote teaching

## Abstract

Paid full-time summer undergraduate research programs (SURPs) increase persistence in science, technology, engineering and math (STEM) degrees and later careers. Research disruptions during the summer of 2020 due to the COVID-19 pandemic required a transition to Emergency Remote Teaching (ERT) and created the opportunity to explore whether online-only SURPs were feasible. Data science projects emerged as a particularly feasible option for both mentors and students. A total of 65 students working with 49 mentors employed at five different research sites matriculated into a 10-week full-time paid SURP in 2020, with most projects focused on data science. Program implementation and impact were explored using online student surveys before (n = 62) and after (n = 56) participation, interviews with students conducted after the program ended (n = 10), online surveys of mentors conducted after the program ended (n = 35), and data on persistence in relevant fields. Scholars reported satisfaction with the program and described how the program developed their scientific skills and interest in data science. Mentors surveyed reported that they would be willing to invite another undergraduate student to perform research under their direction using a distance model. About half of the mentors reported that mentoring students online took about the same amount of time as mentoring in-person. Students who completed the program in-person in 2019 and those who participated in the remote program in 2020 had similar early career trajectories, with approximately 60% of both cohorts remaining in related fields five to six years later. Our experience suggests that an online SURP can be feasibly implemented across multiple sites, with positive impacts on student interest in data science.

## 1. Introduction

Undergraduate research increases persistence in science, technology, engineering and math (STEM) degrees and develops key career skills, while being a prerequisite for admission into graduate school (Kuh, 2008). Summer undergraduate research programs (SURPs), which pay students to focus exclusively on research for an extended time, are considered particularly valuable and are thus common offerings in academic, industry and government research settings.

During the summer of 2020, many research organizations greatly limited on-site work to foster social distancing during the COVID-19 pandemic, which meant that SURPs were either canceled or required major changes to program implementation under an Emergency Remote Teaching (ERT) Framework. Our study focused on one statewide SURP, supported by the Delaware IDeA Network of Biomedical Research Excellence (DE INBRE), which opted to continue operating during the summer of 2020 in a mostly online format as a significant proportion of its faculty mentor pool was able and willing to adapt their research projects to those achievable by undergraduates working remotely.

### 1.1. Literature Review

#### 1.1.1. Undergraduate Research as Educational Best Practice

Authentic research experiences are valuable as they allow students to see how well-controlled scientific inquiry is done and enable understanding of its rewards and difficulties (Linn et al., 2015). Full-time summer undergraduate research experiences may be particularly impactful, as students who complete substantive research experiences as undergraduates are more likely to enroll in graduate school as they have a realistic view of the profession (Altman et al., 2019; Vincent-Ruz et al., 2018). These experiences also give bachelors’ graduates “a leg up” in the job market due to their prior real-world laboratory experience. Equally important is the recognition that not all students will find research to their taste, and early research experiences help these students identify other careers that do not involve research in time to maximize the effectiveness of their undergraduate education (Webb, 2007).

While summer undergraduate research programs (SURPs) are regularly run by academic and other entities nationwide (National Academies of Sciences, Engineering, and Medicine [NASEM], 2017), these programs are traditionally offered in-person. The COVID-19 pandemic required programs to make changes to implementation, up to and including cancelation. Understanding the implementation challenges, successes, and impact of these online programs can help the field both continue to offer these experiences during future pandemics and also identify opportunities to improve the quality and impact of online and in-person SURPs in the future. One previous study of remote SURPs offered during COVID identified gaps in faculty experience mentoring students remotely (Erickson et al., 2022). Other studies showed that remote experiences were reasonable and often feasible to implement, especially when sufficient time is dedicated to planning (Hess et al., 2023; Grineski et al., 2022).

#### 1.1.2. Theoretical Framework: Emergency Remote Teaching (ERT)

Emergency Remote Teaching (ERT) refers to temporary and rapid shifts in instruction from traditional, primarily face-to-face to remote delivery due to an unplanned crisis (Hodges et al., 2020). It differs from other types of remote learning that are often pre-planned or more intentional in design. Certainly, the COVID-19 pandemic resulted in the use of ERT across a variety of educational settings. Key constructs of ERT include (a) Timing: the crisis is emergent and adaptation must occur quickly; (b) Focus on continuity: one main goal is to maintain student access to learning rather than develop a comprehensive replacement for face-to-face education; and (c) Constrained planning and design: given the timing constraints, ERT can lack more robust planning processes and approaches and must rely on available technology and tools.

This study uses the ERT framework to explore how a SURP that was normally offered in-person was successfully translated into the remote environment during the COVID-19 pandemic. Key ERT constructs will be used to explain the changes and their rationale.

### 1.2. Delaware INBRE Context

The Delaware IDeA Network of Biomedical Research Excellence (DE INBRE) is a statewide program supported by the National Institutes of Health (NIH) and the State of Delaware whose mission is to build the infrastructure and workforce necessary to foster biomedical research. In 2020, it was composed of five partner institutions, ranging from a large, very high research activity doctoral university to a state-wide community college system, and included two health system partners. The network also includes six affiliate organizations focused on fostering Delaware’s public health and its biotechnology industry (see Table 1).

Since its founding in 2002, DE INBRE has run a highly successful in-person SURP serving undergraduate students who are either residents of the State of Delaware or enrolled in a Delaware institution of higher education. In a typical year, DE INBRE’s SURP serves approximately 70 students (“Scholars”) who are placed into full-time, 10-week-long research internships under the direction of research-active professors/clinicians based at either a DE INBRE partner or affiliated institution or active researchers affiliated with INBRE programs based in other states.

#### 1.2.1. Emergency Remote Teaching Within DE INBRE SURP

The ERT constructs are a useful organizing principle for describing how the DE INBRE program converted to a virtual program under the emergency context of COVID-19.

##### Timing

In January of 2020, DE INBRE’s SURP had recruited 117 researchers from across Delaware willing to serve as research mentors during summer 2020. Applications opened to students in mid-January 2020, and the program had received 194 applications when the application period closed on 1 March. At that time, the trajectory of COVID-19’s impact on the program was uncertain, so possible scenarios were discussed among program leadership, which supported the possibility of a virtual program while applications were being evaluated.

##### Focus on Continuity

During applicant review in late March 2020, it still seemed possible that the 2020 SURP could be held normally. However, contingency planning began immediately, and a consensus arose that the program should proceed with as many student/mentor pairs as possible, even if the students must work from home, as cancelation would result in participants losing a key research training opportunity, which could negatively shape their career trajectories. As April arrived, the trend suggested that undergraduates would not be allowed onsite at any of the participating research sites, both due to the impact of the pandemic on the research hospitals and state/institution policies for infection containment. Thus, all offers of admission to the 2020 SURP included a statement that the program could still be canceled depending on State mandates. Meanwhile, planning began to pivot the program into a fully online format.

##### Constrained Planning & Design: Opportunity to Focus on Data Science

Mentors who had previously agreed to host a student for summer 2020 were contacted to assess whether the proposed research project was amenable to the student working remotely, and if not, whether the mentor was willing/able to modify the project so that remote work was possible. Program staff worked with mentors to identify potential challenges to the proposed online format and brainstorm how best to pivot projects to tasks that would be feasible to complete remotely. These suggestions included a pivot to bioinformatics or other data science projects, rather than conducting wet lab experiments or other primary data collection. Of the 49 mentors who had been matched with an undergraduate research Scholar for summer 2020, only nine opted to defer and wait to take on the student until they could work in the laboratory “in person” and/or until their COVID-related responsibilities subsided and they had sufficient time for mentorship. Students were also given the option to complete the program virtually or defer their participation until fall 2020 or beyond. Of the 77 students who received offers for the DE INBRE SURP, three declined, a proportion similar to previous years. This matching process resulted in a total of 65 participants in the 2020 DE INBRE Summer Scholar program, four of whom were able to work some hours in-person at a biological laboratory, while the remaining 61 completed the program fully virtually.

In a typical year, the vast majority of SURP projects are either wet laboratory investigations or are “hands-on” work with human subjects, with a small minority of projects (data science, protein/molecular modeling, bioinformatics, image analysis) being primarily or solely computer-based. As the remote model necessitated all projects to be completed on computers at the students’ homes, mentors were surveyed to determine what computer skills their students would need to complete the project, and program staff worked to identify potential challenges to the distance format as well as opportunities to pivot projects to tasks that would better fit the circumstances. As the vast majority of SURP Scholars had little to no prior experience with computer programming, command line computer interfaces, scientific software, or spread sheets, and are life sciences/health professions majors, program staff collaborated with institutional (such as scientific computing support at host institutions, Bioinformatics and Data Science Graduate Student Association, DE INBRE Bioinformatics and Data Science Core Facility) and other entities (including live Software Carpentry workshops given by zoom as well as asynchronous online training available from numerous entities across the world) to identify and/or develop training opportunities in the use of needed scientific software.

### 1.3. Research Questions and Scope

Given the novelty of this transition and the potential for future disruptions that would necessitate ERT, understanding the experience and outcomes of participants in remote SURPs is warranted. Previous research on SURP transitions in the ERT context has suggested that future work focus on following up with students to assess career trajectory and understanding the experience of mentors (Erickson et al., 2022). Given those opportunities to contribute to the literature, the following research questions guided this work:

1. To what extent does participating in the program within an ERT context influence students’ skills, interest and confidence with data science approaches?
2. How do research mentors perceive the experience of remote mentoring?
3. How do the short-term career trajectories of participating students compare to those who participated in a traditional in-person SURP?

## 2. Methods

### 2.1. Implementation Procedures

As in prior years, 2020 DE INBRE Summer Undergraduate Research Program (SURP) Scholars participated full-time for 10 weeks (37.5 h per week). In response to COVID-19–related restrictions, the program was delivered in a fully remote format and consisted of four core components implemented concurrently (Figure 1).

First, Scholars completed required training in the responsible conduct of research, data security, and the protection of research subjects. Second, Scholars engaged in mentor-directed research activities adapted for remote work, which primarily involved data analysis, literature reviews and evidence synthesis, protocol and tool development (e.g., materials for Institutional Review Board review), and database creation, cleaning, and management. Third, Scholars participated in distance-adapted professional development activities, including a program orientation, a synchronous mid-program workshop focused on scientific communication, and additional required workshops tailored to the needs of individual research sites. Optional professional development opportunities emphasized data science skill development and included training in Unix Shell, R programming, Microsoft Excel, and career development panels offered across partner institutions and national programs.

Finally, Scholars completed a public research dissemination requirement. Rather than presenting at in-person conferences, students presented their work at virtual symposia hosted by their home institutions and/or the University of Delaware. These presentations included virtual posters or oral presentations delivered synchronously or asynchronously, accompanied by opportunities for question-and-answer sessions with mentors, research staff, and peers. Collectively, these components allowed the program to maintain core elements of undergraduate research training while adapting delivery and content to a fully remote, emergency context (Figure 1).

### 2.2. Research Approach

#### 2.2.1. Study Participants

Three groups of participants were involved in this study. First, students who participated in the 2020 SURP (intervention group) were included in surveys and interviews. Second, students who participated in the 2019 SURP, which was fully in-person, were included as a comparison group. Third, mentors who worked with one or more students during the 2020 SURP were included in post-program surveys.

#### 2.2.2. Data Collection Tools and Analysis

Given the novelty of the program, several data gathering approaches were used to explore program implementation and impact. These approaches included online student surveys before and after participation, interviews with students conducted after the program ended, and online surveys of mentors conducted after the program ended.

Student surveys captured demographic data, previous experience with research, perceptions of the impact of the program on professional and scientific skills as well as opportunities for improvement. Survey tools were developed by a professional evaluator, experienced with social science survey research and pilot tested with students in prior years. Additional validity and reliability of the items and instruments were not established. Surveys were administered via Qualtrics and analyzed in Microsoft Excel using descriptive statistics (e.g., frequencies). In order to compare demographic characteristics of the remote program to past (in-person) programs, 2019 student survey data were used and analyzed in a similar fashion. This included similar student surveys and tracking of persistence in biomedically relevant fields.

Alumni tracking of all students included searches of publicly available data (e.g., LinkedIn profiles, websites and other social media) along with actively emailing alumni to document their progression. In fall 2025, all students from 2019 and 2020 were searched to document whether they were enrolled in graduate education (and if so, which field) and/or their current occupation. Academic programs and occupations were coded as either (1) non-science, (2) biomedical or health science (e.g., medicine, nursing, bioinformatics, pharmaceutical research), or (3) other science (e.g., chemist, plant science). One coder assigned one of three categories to each student using an existing coding schema that had been developed for the program in previous years.

Student interviews (n = 10) expanded on responses from surveys and focused on the ways the program impacted their plans for the future. Scholars were chosen for interviews at random and were invited via email. Interviews were completed with the random sample based on student and interviewer availability (the number of selected students who were chosen but were unavailable to be interviewed is not known). Student interviews were recorded and transcribed. Modified thematic analysis (Maguire & Delahunt, 2017) was used to identify common themes across interviews and to choose illustrative quotes to better understand quantitative findings from surveys. Using a modified approach, one coder reviewed all transcripts and generated a preliminary set of themes across interviews. A second coder then reviewed both the preliminary themes and the entire dataset. Two coders agreed on common themes and collaboratively selected illustrative quotes. This approach is modified from traditional thematic analysis, which often includes the use of more specific codes (developed a priori or concurrent with analysis) and double coding. That more complex approach was not used here due to the limited sample size (n = 10).

Short mentor surveys gathered information about the nature of the tasks completed by Scholars, their experience with remote mentoring and suggestions for program improvement. Mentor surveys were analyzed using descriptive statistics, while quotes from open-ended items were used to help contextualize the quantitative findings.

Across data sources, findings were triangulated by looking for similarities and differences across sources. The results are presented with multiple sources combined.

These evaluation procedures were reviewed and deemed Exempt (#1642383–1) from further review by the Institutional Review Board at the University of Delaware. Participant data were treated confidentially, and participation in the evaluation procedures (e.g., surveys, interviews) was separated from program participation for both students and mentors.

## 3. Results

### 3.1. Description of Program Participants

End-of-summer surveys were collected from 56 (89%) Scholars enrolled in the program. About one-quarter of the 2020 DE INBRE Summer Scholars who responded were first-generation college students, and 18.5% were from low-income families. Less than half (46.4%) were White and 12% were Hispanic or Latino. The race/ethnicity data were similar to that of participants in a 2019 (in-person) experience, while the percentage of students from low-income families was lower in 2020 compared to 2019 (See Table 2).

Similar to the diversity of our partner institutions, students hailed from a broad range of majors and engaged in primarily human subjects research and data science projects. Only five (6.8%) participants in 2020 were majoring in math or computer science; most had life science (40.5%) or health science (24%) majors. In 2019, only 3 participants (3.8%) were majoring in math or computer science, while 12 (15%) hailed from engineering.

### 3.2. RQ #1: To What Extent Does Participating in the Program Within an ERT Context Influence Students’ Skills, Interest and Confidence with Data Science Approaches?

On the survey, all students agreed that the program increased their confidence in their ability to do research; nearly all agreed that the program improved their communication (96%) and organizational skills (96%). According to one student, “*I learned that unexpected things can happen a lot in a short span of time … and*…*. [how to] improvise on the spot so we can make sure we have a decent enough project to be able to present at upcoming conferences*.”

Of the students who had previous research experience (n = 38), over half (53%) reported that they learned a lot more this summer compared to their previous experience. Only 3 of these students (7.9%) reported that they learned a lot less than in their previous experience. These same students reported that they spent more time this summer interacting with their primary mentor (n = 20, 52.6%), more time interacting with others in their lab/research group (n = 18, 47.4%) and participated more in their lab/group meetings (n = 27, 71%) compared to their previous research experience(s).

Scholars reported high levels of satisfaction with the program (all survey respondents said they would recommend the program; 75% rated their satisfaction with the program as a five out of five) and described the benefits of the program on their scientific skills. In interviews, one student described the skills learned, *“I really learned the importance of communication and teamwork. It was very interesting getting to know and working together when we were all very different. I feel like there were a lot of hurdles and jumps with it being online and some of us even being out of state, but I think it really showed how important communication skills, working as a team, leadership qualities, and … making sure we stayed organized using Google Drive [was to our project].”*

#### Data Science Impact

Students reported high levels of engagement with the optional PD offerings; 84% attended one or more workshops offered through the NIH OITE Program; many reported participating in the Unix Shell course (32%) and R programming course (35%). These PD offerings translated to improvements in Scholar interest and skills in data science more broadly. Many students (78%) agreed that the program increased their interest in data science and/or bioinformatics, and the majority agreed that the program improved their skills in data science and/or bioinformatics (82.6%) or database creation and/or management (69%) (See Table 3). One student participant stated, “*I definitely obtained different skills in terms of using data analysis and collection [tools such as] REDCap, Excel, and other [databases] I had never used before, which was beneficial to know for analyzing and interpreting data in the future*.”

### 3.3. RQ #2: How Do Research Mentors Perceive the Experience of Remote Mentoring?

Surveys were collected from 35 mentors (69%), representing 37 Scholars (56%). The overwhelming majority of mentors (94%) reported that they would work with another undergraduate student in their research using a distance model. Several reported that the experience working with students remotely opened their minds to the benefits of this approach: “*It is quite rewarding to find out that physical distance can be bridged nowadays with the enabling technology and the live presentation can be done as effectively. I think in the future of the program, distance or hybrid model can become an option*.”

About half of mentors (55.9%) reported that mentoring students remotely took about the same amount of time as mentoring in-person; only three (8.8%) reported that remote mentoring took up much more time compared to in-person. Several mentors requested additional support on how to effectively mentor students remotely, “*It would be nice to have some advice for virtual mentoring and projects versus in person*”.

### 3.4. RQ #3: How Do the Short-Term Career Trajectories of Participating Students Compare to Those Who Participated in a Traditional In-Person SURP?

To explore the longer-term impact of the program on students, students from both 2019 and 2020 were tracked to understand their academic and career trajectories. Of the students who participated in the program in 2019, current status was available for 69% (55 of 80 students). Of those former participants, 51% (n = 28) were working in biomedical or health-related fields, and 9% (n = 5) were currently enrolled in graduate school in biomedical or health-related programs. In total, 60% (n = 33) of program alumni from 2019 are engaged in relevant fields in some form. For the 2020 students (n = 65), current data were available for 41 (63%). Of those, 29% (n = 12) are working in biomedical or health-related fields, and another 29% (n = 12) were enrolled in graduate school in biomedical or health-related fields. This suggests that there was little difference in the career trajectory of those who participated in 2019 (60%) compared to the modified version of the program in 2020 (58%).

## 4. Discussion

Importantly, the fully remote format examined in this study was implemented under Emergency Remote Teaching (ERT) conditions, defined by a rapid and unplanned shift to remote delivery in response to a crisis rather than a purposefully designed online learning environment (Bond et al., 2021). The findings should therefore be interpreted in light of the program’s primary goal of maintaining continuity of undergraduate research training during an unprecedented disruption.

While the COVID-19 pandemic will likely have significant and long-lasting effects on academia and research, our experience shows that positive, perhaps unintended, consequences are also likely. By nature of the distance format, most of our Scholars were involved in data science tasks, and the overwhelming majority reported an increased interest and skills in these areas. This observation was notable as the overwhelming majority of 2020 DE INBRE Summer Scholars were either life sciences or health science majors, a group of students who typically have little background or initial interest in quantitative analysis or programming. Given that NIH has made several calls for an increase in data science training and research, these skills may prove to be invaluable to these students. According to the 2018 NIH Strategic Plan for Data Science, “*Data science holds significant potential for accelerating the pace of biomedical research*” (National Institutes of Health, 2018), and expanding the number and background of students with data science training could help fill future gaps in research fields.

Viewed through an Emergency Remote Teaching lens, the observed gains in student confidence, professional skills, and interest in data science are notable, as the program was not designed as an online-first research experience. Rather, data science emerged as an adaptive response to constraints on in-person research activities, suggesting that emergency conditions can catalyze exposure to computational approaches for students who might not otherwise engage with these methods. Future SURP opportunities in data science could be a particularly good fit for the distance model.

Our experience suggests that a fully online SURP can be feasibly implemented across multiple sites, with a positive impact on students. This is consistent with previous studies of COVID-19 disrupted programs, which also demonstrated feasibility (Yang Yowler et al., 2021; Erickson et al., 2022). Mentors were satisfied with the program, and the overwhelming majority would work with another student using a fully remote model. Within the context of Emergency Remote Teaching, mentor reports of comparable time demands and high willingness to mentor remotely in the future suggest that undergraduate research mentoring can be sustained during periods of disruption. At the same time, mentors’ requests for additional guidance on virtual mentoring underscore that ERT models rely heavily on improvisation and may require targeted support to reduce cognitive and administrative load on faculty. Other research on mentor experience during remote SURPs also found high levels of satisfaction, especially in the quality of relationships (Speer et al., 2021). In interviews, Scholars reiterated the themes revealed by the survey data, namely that the experience offered benefits, including skill development and professional networking. Tracking of student alumni from both 2019 and 2020 suggests that the short-term outcomes of participants who engaged in the remote program were not substantially different from those who participated in-person.

In addition, both mentors and Scholars praised the distance model and opened the door to continuing its components even after the pandemic. This was especially true for PD offerings, which had previously been held in person, which required significant travel and coordination time for Scholars and staff who were working across institutions spread across Delaware. Five years later, DE INBRE still largely provides PD training in a synchronous online format. This distance model allowed students across sites to participate in the same workshops, expanding offerings and participation. It has also allowed DE INBRE to more easily share its PD offerings with other SURPs, which further increases their impact.

### 4.1. Limitations

Several limitations should be considered when interpreting these findings. Because the program was delivered under Emergency Remote Teaching conditions, participation was contingent on students having reliable internet access, appropriate technology, and adequate space to work from home. These constraints are characteristic of ERT contexts and likely shaped both participation decisions and student experiences. For one, certain types of research experiences (e.g., clinical experience, bench science) cannot be feasibly implemented via distance learning and thus were not offered to students. Several of the mentors mentioned that students were not able to have patient interactions nor conduct the experiments that would typically be involved in their lab, which is a concern seen in the literature (Iuliano et al., 2021). Future research should consider whether remote versions of these types of experiences can suitably substitute for in-person experiences.

Secondly, our experience suggests that certain student characteristics may be associated with success in these programs. For example, students with prior experience with research, particularly when the summer remote experience is with the same research group, may be more successful with the distance model, perhaps due to established personal connections. Previous research has shown that students with more research experience have higher GPAs and are more likely to continue to post-graduate programs (Chamely-Wiik et al., 2023). Nearly 70% of our summer 2020 participants had some previous research experience; several others without this experience opted to defer their participation until an in-person model was feasible. Students without reliable internet access or quiet study spaces were not able to participate and are thus not included in our data.

Lastly, developing solutions to address the challenges associated with conducting a completely virtual summer program imposed new administrative burdens on the management team and institutional computer support personnel. Coordinating with mentors on needed research project adjustments, staying informed of evolving institutional COVID-19 policies and adapting accordingly, and communicating with network partners led to significant increases in administrative effort. Further, software license restrictions at some research sites prevented software installation on student-owned computers. Thus, computer technology support was needed to set up institution-owned computers that students could log into remotely to run scientific software. In addition, few Scholars had either the academic background or advanced computer skills needed for data science research at the start of the program. Thus, significant information technology support was needed to implement the training workshops necessary for students to develop the skills needed for them to contribute meaningfully to data science-based research projects. While these additional challenges were addressed in the moment, future remote SURPs may require additional or different staff support compared to traditional face-to-face programs.

Our evaluation approach also had several limitations that should be acknowledged in order to contextualize results and identify areas for future study. For one, we did not employ a theoretical framework for the research design or analysis, as this program design resulted from a pivot needed as a response to practical challenges resulting from the COVID pandemic (e.g., ERT). Other theoretical frameworks could be used in the design and evaluation of online SURPs that are implemented more deliberately. In addition, while data from 2019 were used to help understand the ways in which students participating during COVID were different from previous years and to compare their early-stage career trajectory, we did not include a true control group of students who did not participate in any SURP. Future researchers should consider ways to include quasi-experimental or other comparison groups to improve the overall validity of claims and build our understanding of the long-term impact of remote programs operated under ERT conditions. Lastly, the small sample size for the student interviews should be interpreted with caution. We used the interview data to triangulate data from other sources, but the small sample size limits the generalizability of the qualitative findings.

### 4.2. Conclusions and Future Directions

Despite the limitations, this experience illustrated that flexibility in responding to challenges can result in an impactful program that meets the needs of mentors and mentees alike. Certain types of research experiences, especially those focused on data science applications, might be a particularly good fit for remote experiences, even when in-person programs are available. Empirically, this study extends prior descriptive accounts of remote SURPs (e.g., Erickson et al., 2022) by incorporating multiple data sources and longitudinal alumni tracking, allowing for comparison with a pre-pandemic in-person cohort. In doing so, it provides evidence that continuity of research training—rather than modality alone—may be a critical mechanism supporting persistence in biomedical and health-related fields.

While Emergency Remote Teaching conditions are, by definition, temporary, several adaptations developed during this period may have enduring value. In particular, the remote delivery of professional development activities and the integration of data science-focused research tasks may complement future in-person or hybrid SURP models. However, these findings should not be interpreted as evidence that fully remote undergraduate research experiences can or should replace in-person programs under normal conditions.

Future research should examine how individual program components, including mentor training, professional development, and research task design, can be intentionally structured for remote or hybrid undergraduate research experiences. Research informed by the Emergency Remote Teaching framework could identify which adaptations developed during crisis-driven pivots are most effective for supporting student outcomes. Finally, longer-term alumni tracking is needed to assess how remote and hybrid research experiences influence graduate education and career trajectories.

## Figures and Tables

**Figure 1.**
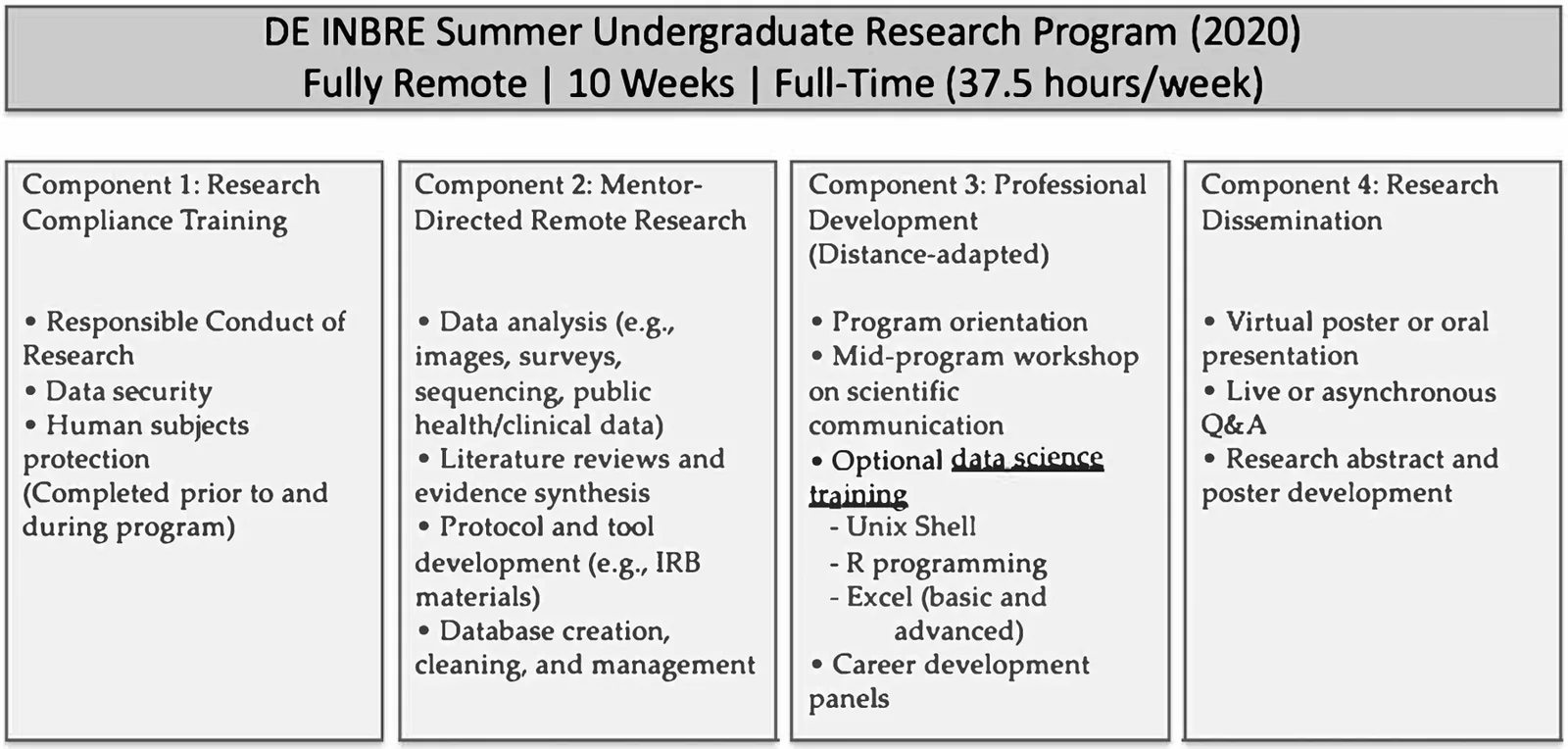
Core components of the DE INBRE Summer Undergraduate Research Program delivered under Emergency Remote Teaching conditions in 2020.

**Table 1.** Description of Delaware INBRE partner organizations.

Institution Name	Type^a^	Other Information
University of Delaware	Very high research activity doctoral university	Lead institution for Delaware INBRE
Delaware State University	High research activity doctoral university	Historically black college/university (HBCU)
Delaware Technical Community College	Associate’s dominant baccalaureate/associate’s college	Statewide community college network with three campuses
Wesley College	Diverse fields baccalaureate college	Private minority serving baccalaureate college
Nemours Children’s Health System	Non-profit children’s hospital and affiliated research enterprise	Pediatric hospital/medical research institute
ChristianaCare Health System	Non-profit acute care hospitals, outpatient clinics and affiliated research enterprise	Major hospital system/health care provider in Northern Delaware with extensive clinical research activities

a Carnegie Classification of Institutions of Higher Education (Indiana University Center for Postsecondary Research, 2017).

**Table 2.** Characteristics of program participants and mentors, summer 2020 and 2019.

	2020 (Total n = 65; Survey Respondents n = 56)		2019 (Total n = 80; Survey Respondents n = 76)	
	Number	Percent	Number	Percent
Student Information				
No prior research experience	17	30.9%	ND^a^	ND
First-generation college student	16	25.4%	28	36.8%
Low-income family	12	18.5%	23	30.3%
Race and Ethnicity				
Hispanic or Latino	7	12.5%	7	9.5%
White	26	46.4%	33	44.6%
Black or African American	15	26.8%	22	29.7%
Asian	10	17.9%	12	16.2%
Other race	5	8.9%	7	9.5%
Major				
Health Sciences	18	24.3%	15	18.8%
Life Sciences	30	40.5%	45	56.3%
Engineering	6	8.1%	12	15.0%
Social Science	4	5.4%	4	5.0%
Math or Computer Science	5	6.8%	3	3.8%
Other	2	2.7%	1	1.3%
**Mentor Information (n = 35)**				
Time spent doing remote mentoring compared to in-person				
Remote much more time than in-person	3	8.8%	-	-
Remote somewhat more time than in-person	6	17.6%	-	-
About the same	19	55.9%	-	-
Remote somewhat or much less time than in-person	6	17.6%	-	-

a ND: no data are available.

**Table 3.** Level of agreement with changes post-program, summer 2020.

	Strongly Agree n (%)	Somewhat Agree n (%)	Somewhat Disagree n (%)	Strongly Disagree n (%)	Not Applicable n (%)
Increased my interest in data science and/or bioinformatics	29 (52.73%)	14 (25.45%)	6 (10.91%)	1 (1.82%)	5 (9.09%)
Increased my confidence in my ability to engage with data science and/or bioinformatics	34 (61.82%)	13 (23.64%)	2 (3.64%)	--	6 (10.91%)
Improved my skills in data science and/or bioinformatics	33 (60.00%)	13 (23.64%)	2 (3.64%)	--	7 (12.73%)
Improved my skills in database creation and/or management	28 (50.91%)	10 (18.18%)	3 (5.45%)	--	14 (25.45%)

## Data Availability

Deidentified data used in this study are available upon request by contacting the corresponding author.

## Abbreviations

The following abbreviations are used in this manuscript:

CITI: Collaborative Institutional Training Initiative
COVID-19: Coronavirus Disease 2019
CV: Curriculum Vitae
DE INBRE: Delaware IDeA Network of Biomedical Research Excellence
ERT: Emergency Remote Teaching
GPAs: Grade Point Averages
HBCU: Historically Black College/University
IDeA: Institutional Development Award
IRB: Institutional Review Board
NIH: National Institutes of Health
NIGMS: National Institute of General Medical Sciences
OITE: Office of Intramural Training and Education
PD: Professional Development
REDCap: Research Electronic Data Capture
STEM: Science, Technology, Engineering, and Mathematics
SURP: Summer Undergraduate Research Program
